# Assessing the Prognostic Significance of Tumor-Infiltrating Lymphocytes in Patients With Melanoma Using Pathologic Features Identified by Natural Language Processing

**DOI:** 10.1001/jamanetworkopen.2021.26337

**Published:** 2021-09-22

**Authors:** Jie Yang, John W. Lian, Yen-Po (Harvey) Chin, Liqin Wang, Anna Lian, George F. Murphy, Li Zhou

**Affiliations:** 1 Division of General Internal Medicine and Primary Care, Department of Medicine, Brigham and Women’s Hospital, Boston, Massachusetts; 2 Harvard Medical School, Boston, Massachusetts; 3 Program in Dermatopathology, Department of Pathology, Brigham and Women’s Hospital, Boston, Massachusetts; 4 Department of Biomedical Informatics, Harvard Medical School, Boston, Massachusetts; 5 Department of Medicine, Brigham and Women’s Hospital, Boston, Massachusetts

## Abstract

**Importance:**

Although tumor-infiltrating lymphocytes (TILs) are an important histopathologic characteristic reflecting host immune response in patients with melanoma, their prognostic value remains controversial. Because manual review of medical records is labor intensive, a survival analysis using a large patient cohort with comprehensive clinical and histopathologic characteristics is lacking.

**Objective:**

To assess the prognostic significance of TILs among patients with cutaneous melanoma using a large cohort established through natural language processing (NLP) algorithms.

**Design, Setting, and Participants:**

This retrospective cohort study analyzed the medical records of 14 436 patients with cutaneous melanoma at Brigham and Women’s Hospital between June 1, 2004, and December 31, 2019. Patients were followed up to death or censored at their last clinical visit.

**Main Outcome and Measures:**

The primary outcome was overall survival (OS). Survival analysis was conducted using Kaplan-Meier curves, the log-rank test, and Cox proportional hazards regression analysis.

**Results:**

A total of 14 436 patients with cutaneous melanoma were identified in the institution’s pathology information system. Using NLP, we established a study cohort of 2624 patients (1462 men [55.7%]; median age, 61 years [interquartile range, 50-72 years]) who had vertical growth phase melanoma with TIL status scored. Absent TILs were identified in 434 patients (16.5%), nonbrisk TILs in 1916 patients (73.0%), and brisk TILs in 274 patients (10.4%). The 5-year survival rate was 71.0% (95% CI, 65.5%-76.9%) among patients with an absence of TILs, 73.8% (95% CI, 71.1%-76.5%) among patients with nonbrisk TILs, and 85.2% (95% CI, 80.0%-90.7%) among patients with brisk TILs. Brisk TILs were significantly associated with improved OS (adjusted hazard ratio, 0.63; 95% CI, 0.42-0.95; *P* = .03; 14.2% OS advantage at 5 years), and nonbrisk TILs were not associated with improved OS (adjusted hazard ratio, 0.87; 95% CI, 0.68-1.11; *P* = .25), compared with the absence of TILs.

**Conclusions and Relevance:**

This study provides evidence based on a large patient cohort from a single institution that suggests that brisk TILs represent an independent prognostic factor for OS among patients with primary cutaneous melanoma. The study also suggests that NLP is a highly efficient tool to facilitate large-scale analyses that involve free-text clinical data.

## Key Points

**Question:**

Are tumor-infiltrating lymphocytes (TILs) an independent prognostic factor for overall survival among patients with primary cutaneous melanoma?

**Findings:**

This cohort study identified clinical and histopathologic characteristics of 14 436 patients with cutaneous melanoma using a natural language processing algorithm to establish a study cohort of 2624 patients with vertical growth phase melanoma and TIL grade. Kaplan-Meier survival analysis and multivariable analysis showed that brisk TILs were significantly associated with improved overall survival (14% advantage at 5 years) compared with the absence of TILs.

**Meaning:**

This study suggests that brisk TILs represent an independent prognostic factor for the overall survival of patients with primary cutaneous melanoma.

## Teaser

This cohort study assesses the prognostic significance of tumor-infiltrating lymphocytes among patients with cutaneous melanoma using a large cohort established through natural language processing algorithms.

## Introduction

Despite recent advances, melanoma remains one of the most aggressive and therapy-resistant cancers, with incidence and mortality rates remaining high.^1,2,3^ In the United States, more than 100 000 new cases of melanoma are diagnosed annually, and almost 7000 people die of the disease.^3^ Even though novel immunotherapies have been proposed and have significantly affected the prognosis for patients with melanoma,^4,5^ reliable biomarkers are still critical in the treatment paradigm because they play a pivotal role in monitoring immunotherapeutic responses and adverse immune reactions.^6^ Tumor-infiltrating lymphocytes (TILs), which are a biomarker regarded as a proxy for the local host immunologic response, have been actively investigated in the past few decades owing to their potential to serve as an independent prognostic factor in melanoma.^7^ Tumor-infiltrating lymphocytes were previously classified as *absent* (lymphocytes are absent or they do not infiltrate the tumor); *present, nonbrisk* (lymphocytes infiltrate only focally and not along the entire base of the invasive component); or *present, brisk* (lymphocytes infiltrate diffusely the entire invasive component or across the entire base of the vertical growth phase).^8,9,10,11,12^ The prognostic value of TILs has varied across different studies and remains controversial.^8,9,13,14^ Clemente et al^15^ reported a 5-year survival rate of 77% for patients with melanoma and with TILs compared with 37% for patients with mealnoma but without TILs. Other researchers have found an association between the presence of significant TILs and sentinel lymph node negativity.^16,17^ On the other hand, Thomas et al^8^ found that the presence of TILs was associated with lower cancer staging, but they also discovered that melanoma-related death rate was lower among patients without TILs compared with those with TILs (30% vs 80%). There also have been studies that demonstrated no association between overall survival (OS) and TILs.^18,19^ Moreover, a study conducted by Sinnamon et al^20^ showed that the prognostic significance of TILs regarding sentinel lymph node positivity in patients with melanoma is more relevant to men than to women, highlighting that the prognostic value of TILs may vary among patients with different demographic characteristics. The inconsistent findings from different studies on the prognostic value of TILs in melanoma also may be associated with small sample sizes, the heterogeneity of the scoring methods used, the inconsistent cutoff of biomarkers, and the limited number of histopathologic characteristics considered.^21^

To establish a well-curated, comprehensive, and large data set to investigate the prognostic significance of TILs, a labor-intensive manual review of electronic health records is often needed owing to a significant number of free-text narrative reports that are not immediately translatable for data analysis. Natural language processing (NLP), which enables computers to process the free-text sections automatically using knowledge-based approaches and/or machine learning algorithms, has shown potential in identifying primary melanoma cases from electronic health records and extracting histopathologic characteristics from pathology reports.^22^ Previous studies have demonstrated that NLP is an effective approach for case identification and may reduce the number of manual reviews in oncology^23,24^ and other medical domains.^25,26^ In this study, we aimed to assess the prognostic significance of TILs in patients with cutaneous melanoma using a large patient cohort with clinical and histopathologic characteristics identified using NLP techniques.

## Methods

### Clinical Setting and Patient Population

**Figure 1.**
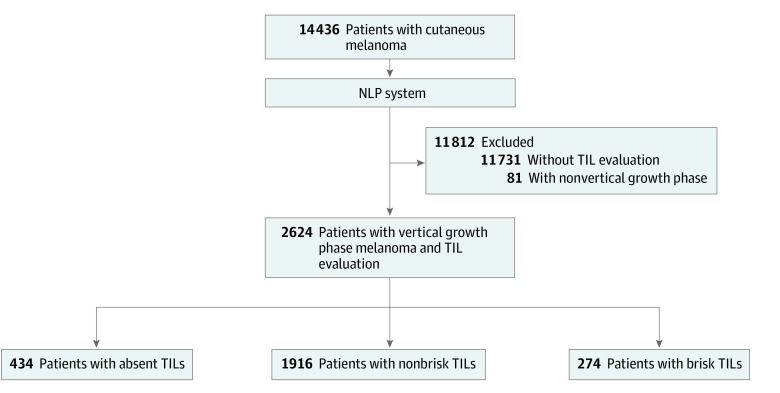
Patient Cohort Selection Process NLP indicates natural language processing; TILs, tumor-infiltrating lymphocytes.

This study was conducted at Brigham and Women’s Hospital, a primary teaching hospital of Harvard Medical School, Boston, Massachusetts. First, we identified patients with primary cutaneous melanoma using pathology reports stored in the institution’s pathology laboratory information system (Sunquest PowerPath; Sunquest Information Systems Inc) between June 1, 2004, and December 31, 2019. Then, we used NLP to process pathology reports to exclusively include patients who met the following 2 criteria in the data analysis: (1) primary invasive melanomas with TIL grade assessed and (2) documentation of vertical growth phase, as shown in Figure 1. For patients with multiple pathology reports, only the most recent synoptic report was included in our study. This study followed the Strengthening the Reporting of Observational Studies in Epidemiology (STROBE) reporting guideline and was conducted with approval by the institutional review board of Mass General Brigham, which waived the informed consent requirement from study participants because of secondary use of hospital safety reports. Patient sex, race, and ethnicity were derived from the institutions’ electronic health record system.

### Extraction of Pathologic Characteristics From Pathology Reports Using NLP Algorithms

The pathology reports at our institution were documented in a semistructured format, with predefined section headers (eg, “Specimen[s]” and “Results”). First, a pattern-matching approach was used to identify report segments containing demographic and pathologic characteristics. Then, a rule-based NLP algorithm was developed to conduct feature extraction. Extracted demographic and report information included patient identifier, date of birth, sex, and report date. Seven pathologic characteristics were extracted: TIL grade, Breslow thickness, mitotic rate, ulceration, histologic regression, microsatellites, and vascular or lymphatic invasion. The findings for each feature were normalized into groups (eg, for TIL grade, “present [brisk]” and “present, at least brisk” were normalized as “brisk”). The NLP algorithm details are described in the eMethods in the Supplement. We used a rule-based approach, instead of machine learning, for the following reasons: (1) the pathology reports contained relatively well-defined, domain-specific terms and patterns; and (2) machine learning methods require manual annotation, which is labor intensive and time-consuming. The rules were developed and fine-tuned based on the feedback from detailed manual review via an iterative process. The source code of our NLP algorithm was released on GitHub.^27^ To evaluate the performance of our NLP algorithm, we randomly selected 200 pathology reports from the entire pathology report data set. Those reports were then manually reviewed by a dermatopathologist to construct a test data set. We used this test data set to assess the NLP system on 6 pathologic characteristics in terms of accuracy, precision, recall, and F1 score. The evaluation metrics are defined as follows: (1) accuracy = number of reports with pathology results correctly extracted by NLP/number of all evaluated reports, (2) precision = number of reports with correctly identified pathology results by NLP/number of reports with pathology results identified by NLP, (3) recall = number of reports with correctly identified pathology results by NLP/number of reports with pathology results, and (4) F1 score = 2 × precision × recall/(precision + recall).

Detailed results on the extraction of the pathological characteristics are shown in the eTable in the Supplement. Overall, our NLP algorithm achieved 99.5% accuracy, 100% precision, 97.7% recall, and a 98.8% F1 score in pathology feature extraction. The workflow to identify and extract patient demographics and pathologic characteristics from free-text pathology reports with our NLP algorithm is demonstrated in eFigure 1 in the Supplement.

### Collection of Demographic and Survival Data

The pathology reports were linked with patients’ electronic health record data to obtain additional demographic information (including race and ethnicity) and death information. Overall survival was calculated from the date of diagnosis to the date of death (due to any cause), the last follow-up date, or December 31, 2019. The study cohort was also linked to Massachusetts death certificate files between 2004 and 2019 to determine the date of death, which helped to identify additional decedents.

### Statistical Analysis

Associations of demographic and pathologic characteristics were examined in context of the 3 TIL grades (ie, absent, nonbrisk, and brisk). Groups were compared using the Kruskal-Wallis test for continuous variables and the χ^2^ test for categorical variables (the Fisher exact test was applied when sample size was <5). Survival was estimated using the Kaplan-Meier method, and the comparisons between different TIL grades were completed using the log-rank test. We applied univariable and multivariable Cox proportional hazard regression models to determine hazard ratios (HRs). Multivariable analysis was performed with the adjustment for all clinical and histopathologic variables collected, including age, sex, Breslow thickness, mitotic rate, histologic regression, microsatellites, and vascular or lymphatic invasion. For exploratory analysis, Bonferroni correction was applied to handle potential type I error during multiple comparisons.^28^ All 95% CIs were 2-sided. The median follow-up was calculated by the reverse Kaplan-Meier method.^29^ The Kruskal-Wallis test was performed using SciPy, version 1.1 (The SciPy community), and all other statistical analyses were performed using R, version 3.6 (R Group for Statistical Computing). All of the tests were 2-tailed and *P* < .05 was considered statistically significant.

## Results

### Characteristics of the Study Cohort

A total of 22 098 pathology reports of 14 436 patients with cutaneous melanoma were identified. After the original cohort had been processed by the NLP algorithm, the final study cohort comprised 2624 patients (1462 men [55.7%] and 1162 women [44.3%]; median age, 61 years [interquartile range, 50-72 years]) whose pathology reports included a vertical growth phase melanoma and a TIL grade evaluation result. The demographic and pathologic characteristics of the study cohort and each TIL grade are shown in Table 1. The median follow-up time was 3.1 years (interquartile range [IQR], 1.0-5.8 years), with a total of 507 deaths (19.3%). The median Breslow thickness was 1.2 mm (IQR, 0.7-2.5 mm). Absent TILs were identified in 434 patients (16.5%), nonbrisk TILs in 1916 patients (73.0%), and brisk TILs in 274 patients (10.4%). For patients with brisk TILs, the median patient age was 58 years (IQR, 47-67 years), which was slightly younger than patients with nonbrisk TILs (median age, 61 years [IQR, 50-72 years]) and patients with absent TILs (median age, 63 years [IQR, 52-72 years]). Among patients with brisk TILs, more than half the cases (n = 158 [57.7%]) were classified as American Joint Committee on Cancer (AJCC) stage T1. This proportion was significantly higher than among patients with nonbrisk TILs (n = 759 [39.6%]) and patients with absent TILs (n = 165 [38.0%]).

**Table 1.** Demographic and Pathologic Characteristics of the Study Population and Their Differences in 3 TIL Grades

Characteristic	No. (%)				*P* value^a^
	Total (N = 2624)	TIL grade			
		Absent (n = 434)	Nonbrisk (n = 1916)	Brisk (n = 274)	
Age, median (IQR), y	61 (50-72)	63 (52-72)	61 (50-72)	58 (47-67)	.002
<50	642 (24.5)	90 (20.7)	472 (24.6)	80 (29.2)	.009
50-69	1258 (47.9)	219 (50.5)	899 (46.9)	140 (51.1)	
≥70	724 (27.6)	125 (28.8)	545 (28.4)	54 (19.7)	
Sex					
Female	1162 (44.3)	200 (46.1)	829 (43.3)	133 (48.5)	.18
Male	1462 (55.7)	234 (53.9)	1087 (56.7)	141 (51.5)	
Breslow thickness, median (IQR), mm^b^	1.2 (0.7-2.5)	1.3 (0.7-2.9)	1.2 (0.7-2.6)	0.9 (0.6-1.5)	<.001
≤1 (T1)	1082 (41.2)	165 (38.0)	759 (39.6)	158 (57.7)	<.001
1.01 to 2 (T2)	630 (24.0)	103 (23.7)	461 (24.1)	66 (24.1)	
2.01 to 4 (T3)	423 (16.1)	67 (15.4)	325 (17.0)	31 (11.3)	
>4 (T4)	403 (15.4)	83 (19.1)	303 (15.8)	17 (6.2)	
Unknown	86 (3.3)	16 (3.7)	68 (3.5)	2 (0.7)	
Mitotic rate, median (IQR)	2 (1-6)	2 (1-6)	2 (1-6)	1 (0-3)	<.001
Ulceration					
Absent	2040 (77.7)	352 (81.1)	1448 (75.6)	240 (87.6)	<.001
Present	536 (20.4)	76 (17.5)	427 (22.3)	33 (12.0)	
Unknown	48 (1.8)	6 (1.4)	41 (2.1)	1 (0.4)	
Histological regression					
Absent	1969 (75.0)	381 (87.8)	1439 (75.1)	149 (54.4)	<.001
Present	628 (23.9)	49 (11.3)	457 (23.9)	122 (44.5)	
Unknown	27 (1.0)	4 (0.9)	20 (1.0)	3 (1.1)	
Microscopic satellites					
Absent	1387 (52.9)	225 (51.8)	1003 (52.3)	159 (58.0)	.007
Present	65 (2.5)	15 (3.5)	50 (2.6)	0 (0.0)	
Unknown	1172 (44.7)	194 (44.7)	863 (45.0)	115 (42.0)	
Vascular/lymphatic invasion					
Absent	177 (6.7)	56 (12.9)	112 (5.8)	9 (3.3)	<.001
Present	1468 (55.9)	250 (57.6)	1092 (57.0)	126 (46.0)	
Unknown	979 (37.3)	128 (29.5)	712 (37.2)	139 (50.7)	

Abbreviations: IQR, interquartile range; TIL, tumor-infiltrating lymphocyte.

^a^ The Kruskal-Wallis rank test was used for continuous variables, the χ^2^ test was used for sex and vascular or lymphatic invasion, and the Fisher exact test was used for the characteristics with fewer than 5 patients (ie, Breslow thickness category, ulceration, histologic regression, and microscopic satellites).

^b^ Based on American Joint Committee on Cancer staging: T1, Breslow thickness 1.0 mm or less; T2, Breslow thickness 1.01 to 2.0 mm; T3, Breslow thickness 2.01 to 4.0 mm; and T4, Breslow thickness greater than 4.0 mm.

### Risk Factors Associated With OS

The median follow-up time was 3.1 years (IQR, 1.0-5.8 years), and there were a total of 507 deaths (19.3%). The 5-year survival rate for the entire cohort was 74.3% (95% CI, 72.1%-76.5%). The 5-year survival rate was 71.0% (95% CI, 65.5%-76.9%) for patients with absent TILs, 73.8% (95% CI, 71.1%-76.5%) for patients with nonbrisk TILs, and 85.2% (95% CI, 80.0%-90.7%) for patients with brisk TILs.

**Figure 2.**
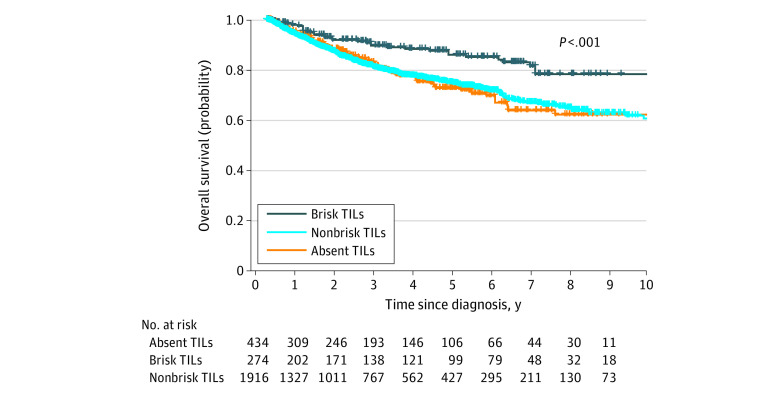
Kaplan-Meier Survival Curve for All Patients TILs indicate tumor-infiltrating lymphocytes.

Based on Kaplan-Meier and log-rank survival analysis, brisk TILs showed a statistically significant association with improved OS among all patients (Figure 2). For exploratory analysis, Kaplan-Meier survival analysis for patient subgroups categorized by sex and by Breslow thickness stage (T1-T4) are shown in eFigure 2 and eFigure 3 in the Supplement, respectively.

The results of the univariable and multivariable OS analyses are shown in Table 2. The univariable analyses showed that younger age, female sex, lower Breslow thickness, lower mitotic rate, brisk TILs, absence of ulceration, absence of microscopic satellites, and absence of vascular or lymphatic invasion were significantly associated with increased OS. The multivariable analysis was applied to examine all clinical and histopathologic characteristics. After adjustments for age, sex, Breslow thickness, mitotic rate, histologic regression, microsatellites, and vascular or lymphatic invasion, brisk TILs remained an independent prognostic factor associated with OS (adjusted HR, 0.63; 95% CI, 0.42-0.95; *P* = .03), with a 14.2% OS advantage at 5 years compared with the absence of TILs. On the other hand, nonbrisk TILs did not demonstrate a significant survival advantage (adjusted HR, 0.87; 95% CI, 0.68-1.11; *P* = .25) compared with the absence of TILs. In addition, older age (adjusted HR, 3.37 [95% CI, 2.48-4.58]; *P* < .001), male sex (adjusted HR, 1.50 [95% CI, 1.23-1.82]; *P* < .001), greater Breslow thickness (adjusted HR, 1.05 [95% CI, 1.03-1.08]; *P* < .001), higher mitotic rate (adjusted HR, 1.02 [95% CI, 1.01-1.03]; *P* < .001), presence of ulceration (adjusted HR, 2.14 [95% CI, 1.70-2.70]; *P* < .001), presence of microscopic satellites (adjusted HR, 1.88 [95% CI, 1.23-2.87]; *P* = .004), and presence of vascular/lymphatic invasion (adjusted HR, 1.58 [95% CI, 1.05-2.38]; *P* = .03) were independently associated with increased risk for mortality in our study cohort.

**Table 2.** Risk Factors Associated With Survival Among Patients With Vertical Growth Phase Melanoma

Variable	Univariable analysis		Multivariable analysis^a^	
	HR (95% CI)	*P* value	Adjusted HR (95% CI)	*P* value
Age, y				
<50	1 [Reference]	NA	1 [Reference]	NA
50-69	1.94 (1.45-2.60)	<.001	1.65 (1.22-2.24)	<.001
≥70	4.29 (3.21-5.75)	<.001	3.37 (2.48-4.58)	<.001
Sex				
Female	1 [Reference]	NA	1 [Reference]	NA
Male	1.77 (1.47-2.13)	<.001	1.50 (1.23-1.82)	<.001
TILs				
Absent	1 [Reference]	NA	1 [Reference]	NA
Brisk	0.51 (0.34-0.75)	<.001	0.63 (0.42-0.95)	.03
Nonbrisk	0.97 (0.77-1.22)	.79	0.87 (0.68-1.11)	.25
Breslow thickness, mm^b^	1.11 (1.09-1.13)	<.001	1.05 (1.03-1.08)	<.001
≤1 (T1)	1 [Reference]	NA	NA	NA
1.01 to 2 (T2)	1.81 (1.38-2.38)	<.001	NA	NA
2.01 to 4 (T3)	3.53 (2.73-4.57)	<.001	NA	NA
>4 (T4)	5.54 (4.33-7.10)	<.001	NA	NA
Unknown	2.51 (1.59-3.98)	<.001	NA	NA
Mitotic rate	1.03 (1.03-1.04)	<.001	1.02 (1.01-1.03)	<.001
Ulceration				
Absent	1 [Reference]	NA	1 [Reference]	NA
Present	3.34 (2.78-4.01)	<.001	2.14 (1.70-2.70)	<.001
Unknown	2.22 (1.22-4.06)	.009	2.09 (1.07-4.09)	.03
Histologic regression				
Absent	1 [Reference]	NA	NA	NA
Present	0.97 (0.79-1.18)	.75	NA	NA
Unknown	1.02 (0.42-2.47)	.96	NA	NA
Microscopic satellites				
Absent	1 [Reference]	NA	1 [Reference]	NA
Present	3.48 (2.37-5.12)	<.001	1.88 (1.23-2.87)	.004
Unknown	0.82 (0.68-0.99)	.04	0.86 (0.71-1.05)	.14
Vascular or lymphatic invasion				
Absent	1 [Reference]	NA	1 [Reference]	NA
Present	1.77 (1.19-2.65)	.005	1.58 (1.05-2.38)	.03
Unknown	1.41 (0.93-2.12)	.10	1.59 (1.05-2.42)	.03

Abbreviations: HR, hazard ratio; TILs, tumor-infiltrating lymphocytes.

^a^ Of the 2624 patients, 86 patients were excluded owing to unclear invasion depths, and 2538 patients were included in the multivariable analysis.

^b^ Based on American Joint Committee on Cancer staging: T1, Breslow thickness 1.0 mm or less; T2, Breslow thickness 1.01 to 2.0 mm; T3, Breslow thickness 2.01 to 4.0 mm; and T4, Breslow thickness greater than 4.0 mm.

## Discussion

We used NLP algorithms to efficiently and effectively construct a large study cohort from 1 academic medical center of patients with primary cutaneous melanoma to ensure shared diagnostic criteria, threshold, and consistency by diagnosing pathologists. Although there are several other multicenter studies with a sample size of a few thousand patients (eg, 1367 patients reported by Sinnamon et al^20^ and 2845 patients reported by Thomas et al^8^), to our knowledge, the present study has the largest sample size of patients with primary cutaneous melanoma from a single institution used to study the prognostic value of TILs.

As the most aggressive form of common skin cancer, cutaneous melanoma has attracted much attention from the clinical and research community. However, the mortality rate among patients with melanoma has not significantly improved during the past decade.^3^ This fact underscores the importance of novel and robust prognostic biomarkers in addition to current prognosis modalities, including sentinel lymph node status, Breslow thickness, ulceration, and microscopic satellites. Although melanoma is widely considered a highly immunogenic tumor and the potential prognostic value of TILs has long been a focus of interest, whether the significance of TILs is robust enough to be incorporated into the AJCC staging system is still under debate. Fu et al^21^ suggested favorable prognostic significance of the presence of TILs, whereas Weiss et al^30^ proposed that melanomas with nonbrisk TILs, compared with brisk TILs, appear to be more prognostically associated with melanomas with absent TILs. The discordant conclusions from different studies may be owing to the following 4 reasons. First, while a widely accepted TIL reporting system proposed by Clark et al^11^ exists, which classifies TILs into categories of “absent”; “present, non-brisk”; and “present, brisk,” several studies classified TILs only as to “present” or “absent,” assigned a TIL score on a scale of 0 to 3, or used the “mild,” “moderate,” and “marked” system.^17,31,32,33,34^ Second, small sample sizes may hinder the rigor of the conclusions suggested by some researchers.^9,35,36,37^ For example, Balatoni et al^37^ conducted research on a cohort of 30 patients and proposed that a higher grade of TIL is associated with better OS, whereas de Moll et al^9^ studied a cohort of 94 patients and showed that TILs were closely associated with survival only in ulcerated melanomas. Third, heterogeneity among patient populations and selection criteria may increase the difficulty of assessing and comparing different conclusions.^1^ For example, Thomas et al^8^ studied patients with cutaneous melanoma in all stages from the Genes, Environment, and Melanoma study, which included patients from different countries, and concluded that a higher TIL grade is associated with better OS. On the other hand, Barnhill et al^19^ suggested that there was no association between TIL grade and OS based on a study cohort consisting of a majority of patients with thin melanomas (<1.7 mm). Last, some investigators have observed a favorable OS associated with higher-grade TILs using only univariable analysis, but with a loss of the prognostic significance in multivariable analysis, which further complicates interpretation of the prognostic significance of TILs.^18,38^

With that in mind, our study attempted to establish the largest patient cohort to date from a single institution and use the widely accepted TIL classification reporting system to research the topic. To ensure data quality and consistency, our pathology reports were collected from a single, large academic medical center that generated reports through a standardized pathology review process. Because reviewing more than 20 000 pathology reports to generate our present study cohort would require immense amounts of time and human labor, we applied the NLP algorithm during the data set construction phase. Previously, Malke et al^22^ developed an NLP platform that can identify and abstract melanoma primary prognostic factors with a less than 5% error rate compared with manual extraction, resulting in enormous improvement in efficiency. Our NLP algorithm is capable of automatically parsing pathology reports and can extract more than 97.7% of results with 100% precision, thus significantly reducing workload and allowing for large-scale patient analysis to be performed. With the deployment of the NLP algorithm, we successfully established the largest primary cutaneous melanoma cohort to date, to our knowledge, to study the prognostic significance of TILs, with the standardized absent, nonbrisk, and brisk classification and comprehensive comparative histopathologic profiling as additional advantages. We used direct OS data to evaluate the prognostic value of TILs, instead of using sentinel lymph node status as a proxy,^39^ because we believe that survival data are the most sensitive indicator to assess prognostic value rather than any other proxy. Considering that the principles of developing the NLP algorithm used in identifying and extracting melanoma-related data could be expanded to other disease fields, application of NLP algorithms to construct a study cohort may potentially be an effective and efficient approach to a variety of other related issues in human pathology.

Consistent with the AJCC and previous literature, our results showed that younger age, female sex, lower Breslow thickness, lower mitotic rate, brisk TILs, absence of ulceration, absence of microscopic satellites, and absence of vascular or lymphatic invasion were significantly associated with improved OS.^8,17,18,31^ Previous studies have also shown that thin melanomas exhibit a higher incidence of brisk TIL grade^8,39^; this finding was in concordance with the present study in which thin melanomas (<2 mm) were associated with an increased incidence of brisk TILs (13.1% [224 of 1712]) compared with thick melanomas (>4 mm) (4.2% [17 of 403]). We also observed an increase in brisk TIL grades in younger patients, which has also been described by Mandalà et al^38^ and Thomas et al.^8^ This finding may be explained by decreased innate and adaptive immune functions associated with the aging process.^40^

### Limitations

There are several potential limitations to our study. First, this study was conducted at a single tertiary academic center, and some cases were referred from other centers. There is a possibility that our patient population may have represented more advanced stages of melanoma. However, in the present study, 65.2% of the primary melanomas studied (1712 of 2624) were less than or equal to 2 mm in thickness, which is beneficial to the generalizability of our findings. Still, future studies that involve multiple institutions and different patient populations are needed to further validate our methods and findings. Second, because we adopted a retrospective study design involving pathology report review, there may have been a classification bias in TIL staging. However, a previous study conducted by Busam et al^41^ demonstrated that interobserver agreement in TIL categorization is excellent, even among observers with less experience. Nevertheless, prospective studies would be needed to further confirm the true prognostic value of TIL staging. Third, while immune markers and gene expression profiles may also play a role in survival among patients with melanoma, we did not include them in the present study and leave this to future work.

## Conclusions

This study suggests that brisk TILs are an independent prognostic factor for OS among patients with primary cutaneous melanoma. We also showed that NLP is an effective approach for establishing a large patient cohort with detailed histopathologic features for survival analysis. Based on our findings, we suggest that TIL grade be included in future AJCC staging revisions and routinely incorporated in a standardized manner into primary cutaneous melanoma pathology reports.

## Notes

Supplement.
eMethods.
**eTable.** The Performance of Natural Language Processing System in Extracting Pathology Results From Free-Text Pathology Reports **eFigure 1.** Workflow of the Natural Language Processing System in Extracting Demographics and Pathological Characteristics From Free-Text Pathology Reports **eFigure 2.** Overall Survival According to Patient Sex **eFigure 3.** Overall Survival According to Different AJCC Stages
