# Supplementary material for: Assessing the Prognostic Significance of Tumor-Infiltrating Lymphocytes in Patients With Melanoma Using Pathologic Features Identified by Natural Language Processing
Source: JAMA Netw Open. 2021 Sep 22;4(9):e2126337. doi: 10.1001/jamanetworkopen.2021.26337 (PMC8459191; doi:10.1001/jamanetworkopen.2021.26337)
Supplement: Supplement. — eMethods. eTable. The Performance of Natural Language Processing System in Extracting Pathology Results From Free-Text Pathology Reports eFigure 1. Workflow of the Natural Language Processing System in Extracting Demographics and Pathological Characteristics From Free-Text Pathology Reports eFigure 2. Overall Survival According to Patient Sex eFigure 3. Overall Survival According to Different AJCC Stages [file jamanetwopen-e2126337-s001.pdf]

Supplementary Online Content

Yang J, Lian JW, Chin YP, et al. Assessing the prognostic significance of tumor-infiltrating lymphocytes in patients with melanoma using pathologic features identified by natural language processing. *JAMA Netw Open.* 2021;4(9):e2126337. doi:10.1001/jamanetworkopen.2021.26337

eMethods.
eTable. The Performance of Natural Language Processing System in Extracting Pathology Results From Free-Text Pathology Reports
eFigure 1. Workflow of the Natural Language Processing System in Extracting Demographics and Pathological Characteristics From Free-Text Pathology Reports
eFigure 2. Overall Survival According to Patient Sex
eFigure 3. Overall Survival According to Different AJCC Stages

This supplementary material has been provided by the authors to give readers additional information about their work.

© 2021 Yang J et al. *JAMA Network Open.*

eMethods.

eFigure 1 shows the workflow of our natural language processing (NLP) system to identify and extract patient demographics and pathological characteristics from free-text pathology reports. First, being part of the NLP system, we developed a sectionization module to divide each pathology report into two sections, one being the demographic section and the other being the pathology result section. For the demographic section, we developed a customized demographic information extraction module based on pattern match technique, which was used to extract patient demographic information, including name, sex, medical record number, date of birth, date of receiving sample, etc. For the pathology result section, a customized pattern match module was developed and used to extract pathological features and values, including tumor-infiltrating lymphocyte (TIL), regression, ulceration, invasive depth, etc. As the pathology findings can be written in various ways (e.g., “PRESENT (BRISK)”, “FOCAL BRISK”, “BRISK”, “Present, brisk”), we further developed a finding normalization module with a supervision from a pathologist which was further used to normalize and consolidate the extracted results.

© 2021 Yang J et al. *JAMA Network Open.*

eTable. The Performance of Natural Language Processing System in Extracting Pathology Results From Free-Text Pathology Reports

**Pathology Results**	**Accuracy (%)**	**Precision (%)**	**Recall (%)***	**F1-score (%)**
TILs	99.5	100	96.3	98.1
Ulceration	99.5	100	96.6	98.3
Histological regression	99.5	100	96.7	98.3
Microscopic satellites	99.5	100	100	100
Vascular/lymphatic invasion	99.5	100	100	100
Mitotic Rate	99.5	100	96.8	98.4
**Overall ** **	**99.5**	**100**	**97.7**	**98.8**

Abbreviations: TIL, tumor-infiltrating lymphocytes

*Among the randomly selected 200 samples, there are 27, 29, 30, 31 samples included the TILs, ulceration, histological regression, and mitotic rate results, respectively. The natural language processing system missed 1 sample for each of the above variable, so the recall is (27-1)/27=96.3%, (29-1)/29=96.6%, (30-1)/30=96.7%, (31-1)/31=96.8% for TIL, ulceration, histological regression, and mitotic rate results, respectively.

** The value of the overall row is the average of all the above six variables.

© 2021 Yang J et al. *JAMA Network Open.*

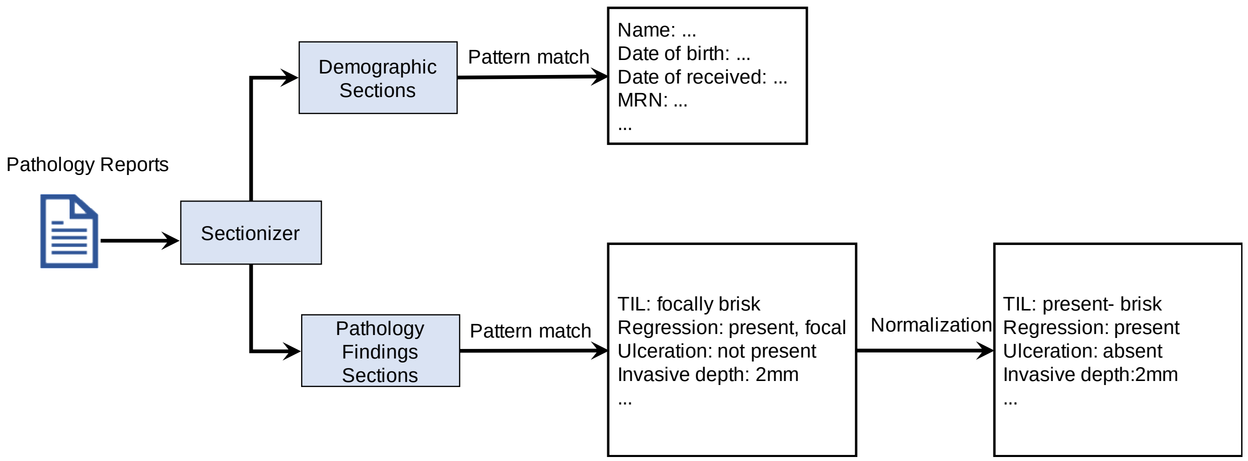

eFigure 1. Workflow of the Natural Language Processing System in Extracting Demographics and Pathological Characteristics From Free-Text Pathology Reports

© 2021 Yang J et al. *JAMA Network Open*.

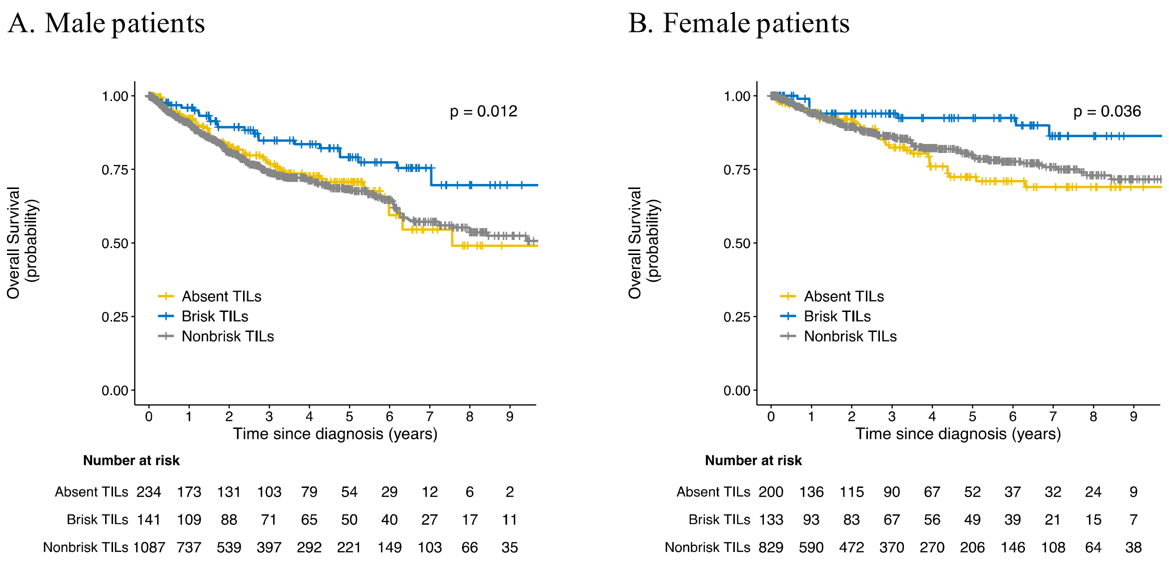

eFigure 2. Overall Survival According to Patient Sex. (A) male patients (B) female patients.

Note: Based on the log-rank test, the brisk TILs showed significant associated with improved the overall survival of male melanoma patients (without adjust other covariables) at Bonferroni-corrected P < .025. The significance in female patients is not robust to Bonferroni correction (P > .025).

© 2021 Yang J et al. *JAMA Network Open.*

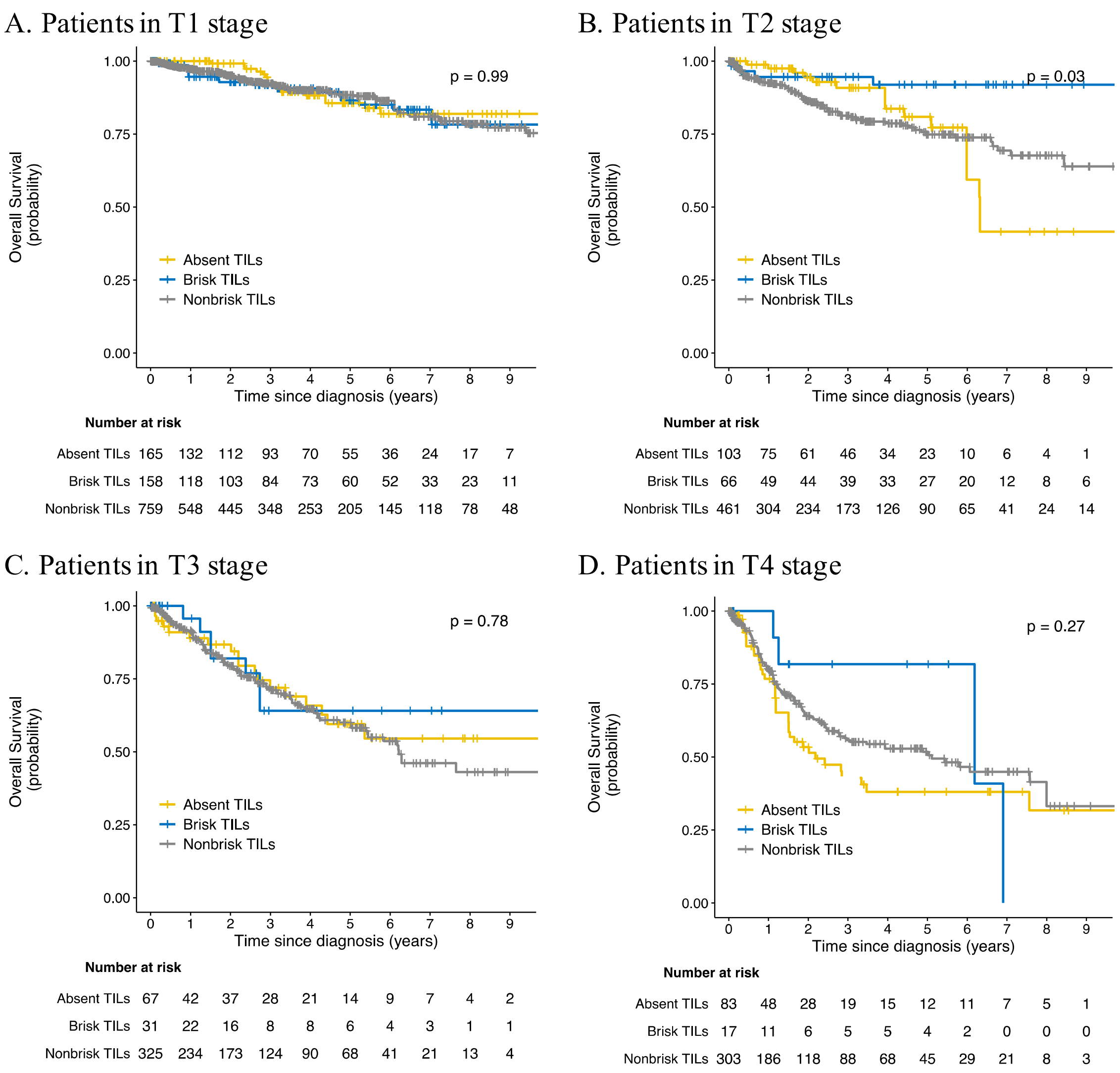

eFigure 3. Overall Survival According to Different AJCC Stages. (A) T1 (B) T2 (C) T3 (D) T4.

Note: Based on the log-rank test, the brisk TILs showed significant associated with improved the overall survival only in the melanoma patients at T2 stage (without adjust other covariables) (P < .05), and the significance is not accepted at a Bonferroni corrected P > .0125. This subgroup analysis has low statistical power due to the limited patients in each group.

© 2021 Yang J et al. *JAMA Network Open.*
